# Three combinations of manual therapy techniques within naprapathy in the treatment of neck and/or back pain: a randomized controlled trial

**DOI:** 10.1186/s12891-016-1030-y

**Published:** 2016-04-23

**Authors:** Kari Paanalahti, Lena W. Holm, Margareta Nordin, Jonas Höijer, Jessica Lyander, Martin Asker, Eva Skillgate

**Affiliations:** Musculoskeletal & Sports Injury Epidemiology Center, Institute of Environmental Medicine, Karolinska Institutet, Box 210, Stockholm, SE-17177 Sweden; Occupational and Industrial Orthopaedic Center (OIOC), NYU Hospital for Joint Diseases, New York University Langone Medical Center, 63 Downing Street, New York, NY 10014 USA; Naprapathögskolan - Scandinavian College of Naprapathic Manual Medicine, Kräftriket 23A, Stockholm, SE-11419 Sweden; Institute of Health Policy, Management and Evaluation, University of Toronto, 4th Floor, 155 College St, Toronto, ON M5T 3 M6 Canada; Unit of Biostatistics, Institute of Environmental Medicine, Karolinska Institutet, Box 210, Stockholm, SE-17177 Sweden

**Keywords:** Muskuloskeletal manipulations, Naprapathy, Back pain, Neck pain

## Abstract

**Background:**

Manual therapy as spinal manipulation, spinal mobilization, stretching and massage are common treatment methods for neck and back pain. The objective was to compare the treatment effect on pain intensity, pain related disability and perceived recovery from a) naprapathic manual therapy (spinal manipulation, spinal mobilization, stretching and massage) to b) naprapathic manual therapy without spinal manipulation and to c) naprapathic manual therapy without stretching for male and female patients seeking care for back and/or neck pain.

**Method:**

Participants were recruited among patients, ages 18–65, seeking care at the educational clinic of Naprapathögskolan - the Scandinavian College of Naprapathic Manual Medicine in Stockholm. The patients (*n* = 1057) were randomized to one of three treatment arms a) manual therapy (i.e. spinal manipulation, spinal mobilization, stretching and massage), b) manual therapy excluding spinal manipulation and c) manual therapy excluding stretching. The primary outcomes were minimal clinically important improvement in pain intensity and pain related disability. Treatments were provided by naprapath students in the seventh semester of eight total semesters. Generalized estimating equations and logistic regression were used to examine the association between the treatments and the outcomes.

**Results:**

At 12 weeks follow-up, 64 % had a minimal clinically important improvement in pain intensity and 42 % in pain related disability. The corresponding chances to be improved at the 52 weeks follow-up were 58 % and 40 % respectively. No systematic differences in effect when excluding spinal manipulation and stretching respectively from the treatment were found over 1 year follow-up, concerning minimal clinically important improvement in pain intensity (*p* = 0.41) and pain related disability (*p* = 0.85) and perceived recovery (*p* = 0.98). Neither were there disparities in effect when male and female patients were analyzed separately.

**Conclusion:**

The effect of manual therapy for male and female patients seeking care for neck and/or back pain at an educational clinic is similar regardless if spinal manipulation or if stretching is excluded from the treatment option.

**Trial registration:**

Current Controlled Trials ISRCTN92249294

## Background

Neck and back pain is common and costly in the general population, and represents an enormous financial burden. The costs stem from loss of work and medical expenses, as well as other direct or indirect expenses [1, 2]. Life time prevalence of spinal pain has been reported to be 54 to 80 % [3]. To manage these painful and disabling conditions manual therapists (i.e. chiropractors, naprapaths, osteopaths, physicians and physiotherapists) often combine manual techniques as spinal manipulation, spinal mobilization, stretching and massage. According to Cochrane reviews, manual therapy appears to be no better or worse than other existing therapies for patients with neck and back pain, and the long term effects are often unclear [4–7]. Other systematic reviews have suggested manual therapy to be an effective and cost effective treatment option for neck pain and back pain [8–10] especially if combined with education and exercise [11].

Rubinstein et al. included in a systematic review a few studies showing that spinal manipulation was more effective for low back pain than mobilization in the short term but not in the long term. Other studies that did not find any difference in effect between the two techniques, and no conclusion was drawn regarding this [12]. Stretching has been suggested to be an effective treatment alternative for low back pain [13] and exercise with combined strengthening, range-of-motion, and flexibility exercises is considered effective for persistent neck pain [14].

According to recent studies naprapathic manual therapy (NMT) is an effective and cost effective treatment alternative for musculoskeletal pain both in the short and the long term [15–18]. Naprapathy in Scandinavia is defined as a system for specific examination, diagnostics, manual treatment and rehabilitation of pain and dysfunction for the musculoskeletal system. NMT is a combination of manual techniques such as spinal manipulation/mobilization, stretching and massage used to treat shortened or pathologic soft and connective tissue that are thought to be common causes to these pain conditions [18]. The profession of naprapathy was initiated in 1907 in the United States, and is today practiced mainly in Finland, Norway, Sweden, and the United States. Since 1994, a naprapath is a registered health professional in Finland and Sweden. Registered naprapaths are overseen by the National Supervisory Authority for Welfare and Health in Finland and by The National Board of Health and Welfare in Sweden. Naprapathy is the largest registered profession within manual therapy in Sweden, larger than the professions of Chiropractic and Osteopathy.

NMT is a young area of research and further studies are needed to develop the discipline and to ascertain quality assurance. Aspects of this are the risk of adverse events and if treatment effects and risks differ between different combinations of NMT (with and without spinal manipulation and stretching). We have performed a three-arm randomized controlled trial (RCT) called the Stockholm MINT-trial (the Stockholm Manual Intervention Trial), where these questions are addressed, in order to ease the consideration between risks and benefits in the choice of treatment strategies for these patients. One study is published based on the first 767 included patients seeking care for non-specific back and/or neck pain in which we found no systematic differences in the risk of adverse events between the treatment arms [19]. Based on the same trial, now with 1057 included patients, we address the following research question: Are there differences in treatment effect on pain intensity, pain related disability and perceived recovery when NMT (spinal manipulation, spinal mobilization, stretching and massage) is compared to NMT without spinal manipulation and, NMT without stretching for male and female patients seeking care for back and/or neck pain?

## Methods

### Design and trial registration

This study is a three-arm randomized controlled trial (RCT), and the trial was registered in Current Controlled Trials (ISRCTN92249294).

### Setting

The treatments were given at the educational clinic of the Naprapathögskolan - Scandinavian College of Naprapathic Manual Medicine, Stockholm, Sweden. The students, who provided the treatments were in their seventh out of eight total semesters, with experience regularly (twice a week) treating patients over a period of five semesters. These treatments include massage and stretching techniques during five semesters, spinal mobilization techniques during three semesters and spinal manipulation techniques during two semesters under supervision of experienced registered naprapaths. The supervision included regular controls of the compliance of the study protocol. The primary investigator and the research team regularly had meetings and education with the students as well as with the supervising naprapaths/teachers. Senior students that participated in the current trial had passed all practical clinical examinations at this level of the education which includes all the manual techniques used.

### Participants

#### Inclusion criteria

The participants eligible for this study were patients, 18–65 years old, seeking care for neck pain (including neck/shoulders and/or upper back, above the 11^th^ thoracic vertebra, with or without pain in upper extremities and chest) and/or back pain (including pain the area below the 10^th^ thoracic vertebra and/or gluteal area with or without pain in lower extremities) and who had not visited the educational clinic during the previous month.

#### Exclusion criteria

1) Not mastering the Swedish language, 2) having scored <2 on a numerical rating scale (0–10) in two questions regarding pain intensity (pain at the present time and the worst pain during the past 4 weeks) in neck and/or back, 3) pregnancy, 4) current or previous cancer, 5) having received treatments for the current complaint by a chiropractor, naprapath, osteopath or physiotherapist during the past month, 6) duration of the current complaint less than 1 week, 7) patient demand/deny spinal manipulation/stretching, 8) contraindication for spinal manipulation according to the Swedish Board of Social Welfare [20], 9) indication for spinal manipulation (hypo mobility in the joints of the spine, with or without pain) not present in the area of complaint, 10) red flags (i.e. previous trauma, inflammatory or rheumatic diseases, drug addict, large rapid weight decrease etc.), 11) specific diagnosis (i.e. ankylosing spondylitis, spinal stenosis, rheumatoid arthritis), 12) sick leave due to planned/completed surgery for neck and/or back pain.

Potential study participants were marked in a ledger at the clinic by a trained receptionist according to inclusion criteria; age, neck and/or back pain and no visit at the educational clinic during the past month. The inclusion process was handled by the supervised and trained student therapists: information about the study to the patients, obtaining of informed consent, the baseline data collection, the decision about inclusion, and the randomization allocation. The therapists as well as the supervising experienced registered naprapaths were thoroughly trained in different aspects of the study protocol at a number of meetings before the start of the study. Uncertainties and questions were discussed in regular weekly meetings during the inclusion period.

### Randomization

A trained research assistant made the randomization in advance and prepared sequentially numbered opaque and sealed envelopes with cards numbered 1, 2 or 3, by drawing these cards from a box. Stratified randomization was used, based on the location of pain: 1) neck and upper back 2) lower back and 3) neck and lower back (pain equally bad in neck and upper back and lower back). For each stratum, randomization in blocks of 99 were performed.

Potential study participants were informed about the study at the first visit. Informed consent was obtained and patients completed the baseline questionnaire suited for the area of pain before the physical examinations and diagnostic assessment by the therapists. The patient and the therapist were unaware of the group assignment until after all baseline data were collected. Treatment allocation was performed by the therapist after the physical examination and the completion of the baseline questionnaire. The therapists were told not to share the result of the randomization to the patient if possible, however this cannot be considered as blinding the patient to the treatment. Each treatment session was scheduled for 45 min.

### Interventions

The interventions in all three arms were conformed to the patients’ condition, however standardized to the greatest extent possible concerning, for example, the length of treatment sessions and how to perform them in different situations, by several education meetings held in advance. The therapists were told to use all manual therapy techniques that were included in the treatment arm according to the randomization protocol, but no other treatment modalities.

1. Naprapathic Manual Therapy (NMT): The therapist was allowed to use all techniques within NMT i.e. spinal manipulation, spinal mobilization, muscle stretching, massage and other soft tissue techniques.
2. NMT excluding spinal manipulation: The therapist was allowed to use all available techniques within NMT except spinal manipulation.
3. NMT excluding muscle stretching: The therapist was allowed to use all techniques within NMT except muscle stretching.

To ensure that the treatments were performed according to the randomization protocol, a random sample of 6 % of the medical records were control post treatment.

### Baseline data collection

The baseline questionnaire was based on questionnaires used in previous studies [18, 21] and includes socio-demographic factors, physical activity, smoking habits, previous pain conditions concerning the current complaint and how the current complaint began, expectations of recovery related to treatment and general health [22]. A modified Chronic Pain Questionnaire (CPQ) was used to assess pain intensity and pain related disability at baseline and at follow-up [23]. The original scale that was based on recall of the past 6 months was changed to the past 4 weeks as we have done in previously published studies [18, 23, 24].

### Follow-up data collection of outcomes

Information of outcomes was collected by self-administrated postal or web-based questionnaires at follow-up, after seven, 12, 26 and 52 weeks. If a patient did not answer or if the questionnaire was not complete, a trained research assistant contacted the patient by phone, mail or letter to remind them, at a maximum of three occasions.

Primary outcomes were pain intensity and pain related disability. Patients graded their pain in three pain questions (current pain, worst pain, average pain) measured with a numeric rating scale, 0–10 (0 = no pain, 10 = pain as bad it could be) with the modified CPQ [23]. Three questions assessed disability and concerned to what degree pain “interfered with your daily activities”, “changed your ability to take part in recreational, social, and family activities,” and “changed your ability to work (including housework)” in the past 4 weeks. The ratings were made on a numeric rating scale, 0–10 (0 = no interference, 10 = unable to carry on with these activities). A mean pain score and a mean disability score was calculated from the questions.

#### Dichotomized primary outcomes

A minimal clinically important improvement (MCI) in pain was defined as a decrease by at least 2 points in pain intensity score at follow-up compared to baseline and a MCI in disability was defined as a decrease by at least 1 point in pain related disability score at follow-up compared to baseline [17, 18, 25–27].

Secondary outcomes were perceived recovery was measured by the question, “*Which of the following statements is most consistent with how you feel your problems in the neck/lower back has changed since you joined this study*?”. The answer alternatives were; 1) Am completely free from pain and have no other complaints originating from neck/ back, 2) Am considerably improved, 3) Am slightly improved, 4) No change, 5) Am slightly worse and 6) Am much worse. For the comparison between groups the answers were dichotomized into recovered (answer alternative 1–2)/not recovered (answer alternative 3–6). Further a question about additional care administered for the neck/back pain the preceding 3 months was included in the follow-up questionnaire.

### Statistical analyzes

Power analyses based on the primary outcomes (a minimal clinical important improvement (MCI) in pain and disability) were performed before the Stockholm MINT trial started, to determine the sample size required. A total of 350 patients per treatment arm, in total 1 050 patients, indicated a power of >80 % to detect a relative risk (RR) of 1.2 to 1.3 for a clinically important improvement in pain and disability in sub group analyses (as neck/back pain, men/women, older/younger) of 150 patients, regarding the primary outcomes.

Analysis was done with an intention to treat approach. To analyze if there were any differences between the treatment arms in MCI in pain and disability and perceived recovery, and to test for confounding, logistic regressions using generalized estimating equations (GEE) were conducted, in all participants and in gender stratified analyses. For further testing if there were any differences in mean pain/disability score, linear regressions using GEE was used taking into account the covariance between repeated measurements of pain and disability, in all participants and in gender stratified analyses [28, 29]. The analysis regarding perceived recovery was performed using ordinal logistic regression with cluster-robust standard errors. All analyses were conducted in the statistical software STATA 13.0.

**Fig. 1:**
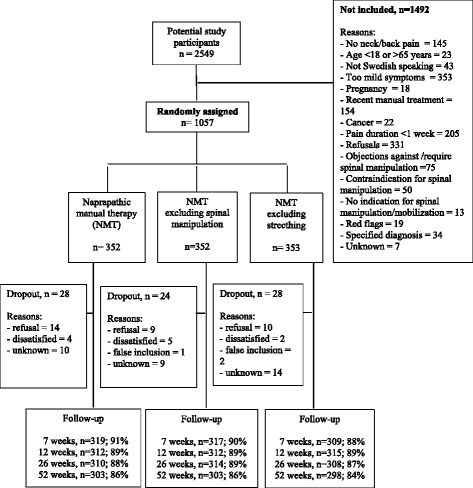
Flowchart of the recruitment, randomization and follow-up

The study population for the analyses of mean scores of pain intensity and mean score pain related disability, and for the analyses of perceived recovery, was 1057 patients seeking care for non-specific back and/or neck pain (Fig. 1). For the analysis of MCI in pain intensity, it was required that, at baseline, the patient should have a pain score of at least 2/10. Therefore, six patients were excluded from these analyses. For the analysis regarding MCI in pain related disability, it was required that the patients should have a disability score at baseline of at least 1/10. Consequently, 286 patients were excluded from these analyses.

## Results

At baseline 1057 patients were randomized to one of three treatment alternatives. The period of recruitment was January 2010 to December 2012 and the follow-up of all patients was finished in January 2014. Figure 1 is a flowchart of the recruitment, randomization and follow-up of patients in the trial. Eighty patients decided to drop-out before the first follow-up; 33 did not want to participate, three were false inclusions, 11 were dissatisfied and 33 dropped out due to unknown reasons. The dropouts were equally distributed between the treatment arms.

Baseline characteristics for the study participants are presented in Table 1. The mean age was 35 years (SD 11.8), and more than 60 % of the patients reported their general health to be very good or excellent. Fifty four percent of the patients sought care for neck pain, 70 % were females and almost 80 % had had experienced similar complaints before. The proportion of patients with chronic pain (>3 months duration) was 36 %. The mean pain at baseline was 5.5/10 (SD 1.8) and the mean disability was 2.6/10 (SD 2.2).

**Table 1:** Baseline characteristics of the study population (*n* = 1057)

	NMT^a^	NMT excluding spinal manipulation	NMT excluding stretching
Characteristics	*n* = 352	*n* = 352	*n* = 353
Mean age (SD)	35 (12.2)	36 (11.9)	36 (11.4)
Sex, %			
Women	68	72	71
Painful area, %			
Back	35	33	33
Neck	54	54	54
Back/Neck	11	13	12
Duration of the			
pain, %			
1 week	17	16	18
2–4 weeks	29	26	26
1–3 months	18	22	21
3–6 months	9	8	7
>6 months	28	28	27
Similar previous complaints,			
%			
Yes	73	79	80
Mean pain intensity at baseline^b^	5.5	5.4	5.5
(SD)	(1.6)	(1.7)	(1.7)
Mean pain related disability at baseline^c^	2.6	2.6	2.6
(SD)	(2.2)	(2.2)	(2.2)
Education, %			
1–9 years	3	4	3
10–12	37	39	37
13–15	50	46	46
>16	11	11	14
General Health, %			
Excellent	14	17	19
Very Good	46	45	42
Good	34	30	34
Somewhat	6	7	4
Bad	1	-	1
Smoking, %			
Daily	18	14	17
Physical activity, %			
on medium or high level^d^	33	33	43
Mean expectations of recovery^e^	6.1	5.9	5.9
(SD)	(3.0)	(2.9)	(2.9)
Previous naprapathic treatment, %			
No	44	41	35
Yes, a few times	30	34	34
Yes, several times	26	26	40
Obesity, %	7	8	7

^a^NMT = Naprapathic Manual Therapy (spinal manipulation/mobilization, stretching and massage)

^b^Pain intensity at baseline was based on three pain items: current pain, worst pain, average pain during the past 4 weeks (NRS 0–10 (0 = no pain, 10 = pain as bad it could be)) and calculated as an average pain score for these items

^c^Disability at baseline was based on three disability items: interference with daily activities, recreational and social activities, and work activities, measured with numeric rating scale, (NRS 0–10 (0 = no interference, 10 = unable to carry on with these activities)) and calculated as an average disability score for these items

^d^Physical activity = medium (effort that would make it difficult to hold a conversation with someone) or high (you have a high pulse, you feel strained and become sweaty) level at least two times/week

^e^Expectations of recovery are measured with NRS 0–10 (0 = not at all likely to be recovered, 10 = very likely to be recovered)

The total number of treatments was 4627 with a mean of 3.6 treatments per patient (SD 1.5). At 12 weeks follow-up 61 % had a MCI in pain intensity, and 41 % in pain related disability. The corresponding chances to be improved at the 52 weeks follow-up were 58 % and 40 % respectively.

None of the characteristics presented in Table 1 turned out to be a confounder in the analyses of the associations between the treatment alternatives and the primary outcomes.

Table 2 presents the odds ratios (OR) with 95 % CI comparing NMT to other treatment arms regarding MCI in pain intensity, pain related disability and perceived recovery at each time point of follow-up. No systematic differences in effect when excluding spinal manipulation and stretching respectively from NMT were found at any follow-up.

**Table 2:** Comparison of the odds of the outcomes between the treatment arms. The odds of having a MCI in pain intensity, MCI in pain related disability and perceived recovery in NMT excluding manipulation and NMT excluding stretching in comparison to NMT, at follow-up after 7, 12, 26 and 52 weeks

MCI	MCI in pain intensity^a^ (*n* = 971)			MCI in pain related disability^b^ (*n* = 691)			Perceived recovery^c^ (*n* = 977)		
Treatment arms	NMT^d^	NMT excluding manipulation	NMT excluding stretching	NMT^d^	NMT excluding manipulation	NMT excluding stretching	NMT^d^	NMT excluding manipulation	NMT excluding stretching
Follow-up in weeks									
		OR 95%CI	OR 95%CI		OR 95%CI	OR 95%CI		OR 95%CI	OR 95%CI
7	1.0	0.93 (0.68–1.27)	1.00 (0.73–1.37)	1.0	1.08 (0.72–1.64)	1.44 (0.93–2.21)	1.0	1.21 (0.88–1.65)	1.12 (0.82–1.53)
12	1.0	0.74 (0.54–1.03)	0.96 (0.70–1.33)	1.0	1.01 (0.65–1.59)	1.38 (0.86–2.20)	1.0	0.90 (0.66–1.23)	0.98 (0.72–1.34)
26	1.0	0.83 (0.60–1.15)	0.85 (0.62–1.17)	1.0	1.21 (0.77–1.91)	1.05 (0.67–1.64)	1.0	0.90 (0.66–1.24)	1.04 (0.76–1.43)
52	1.0	0.84 (0.61–1.17)	0.83 (0.60–1.15)	1.0	0.99 (0.61–1.61)	0.73 (0.46–1.17)	1.0	1.00 (0.73–1.38)	0.89 (0.65–1.22)

^a^MCI (minimal clinically important improvement) in pain intensity was defined as at least two-step decrease from baseline to follow-up, measured with numerical rating scale 0–10 (0 = no pain, 10 = pain as bad as could be)

^b^MCI (minimal clinically important improvement) in disability was defined as at least one-step decrease from baseline to the follow-up, measured with numerical rating scale 0–10 (0 = no interference, 10 = unable to carry on with these activities)

^c^Perceived recovery dichotomized into recovered (= Is completely free from pain and have no other complaints originating from neck/back or Is considerably improved)/not recovered (= Is slightly improved, No change, Is slightly worse or Is much worse)

^d^NMT = Naprapathic manual therapy (spinal manipulation, mobilization, stretching and massage)

**Fig. 2:**
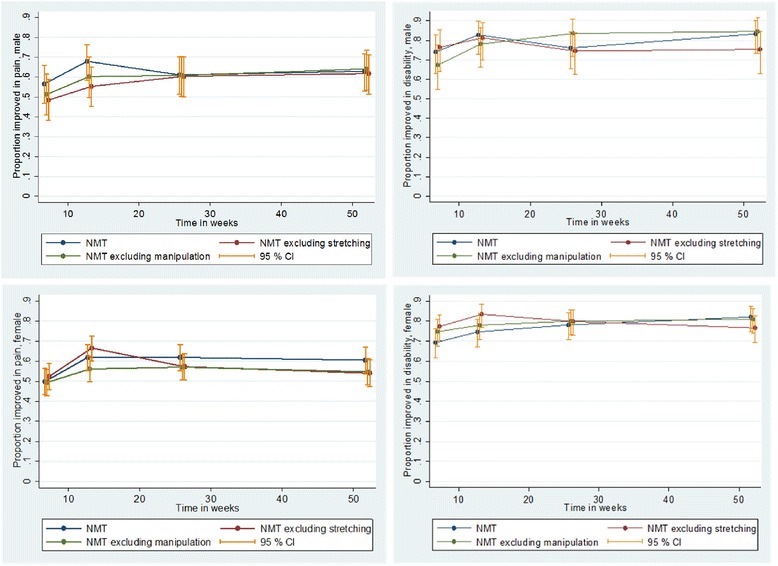
The proportions of patients with MCI in pain intensity and pain related disability. The proportions of male and female patients with a MCI in pain intensity and MCI in pain related disability in the treatment arms with 95 % confidence intervals (95 % CI) at all follow-ups over 1 year

The joint test of difference – in the GEE framework taking repeated measurements at 7, 12, 26 and 52 weeks into consideration – showed no differences in effect between the three treatment arms, concerning the chance to have a MCI in pain intensity (*p* = 0.41; Wald test) and pain related disability (*p* = 0.85; Wald test) during 1 year follow-up (not in table). Further, no disparities were found in gender stratified analyzes of pain intensity (female: *p* = 0.49, male: *p* = 0.61; Wald test) and pain related disability (female: *p* = 0.61, male: *p* = 0.91; Wald test) (not in table). Figure 2 shows the proportion with a MCI in pain intensity and pain related disability with 95 % CI at all follow-ups for males and females separately. No systematic differences in effect were seen when male and female patients were analyzed separately.

**Fig. 3:**
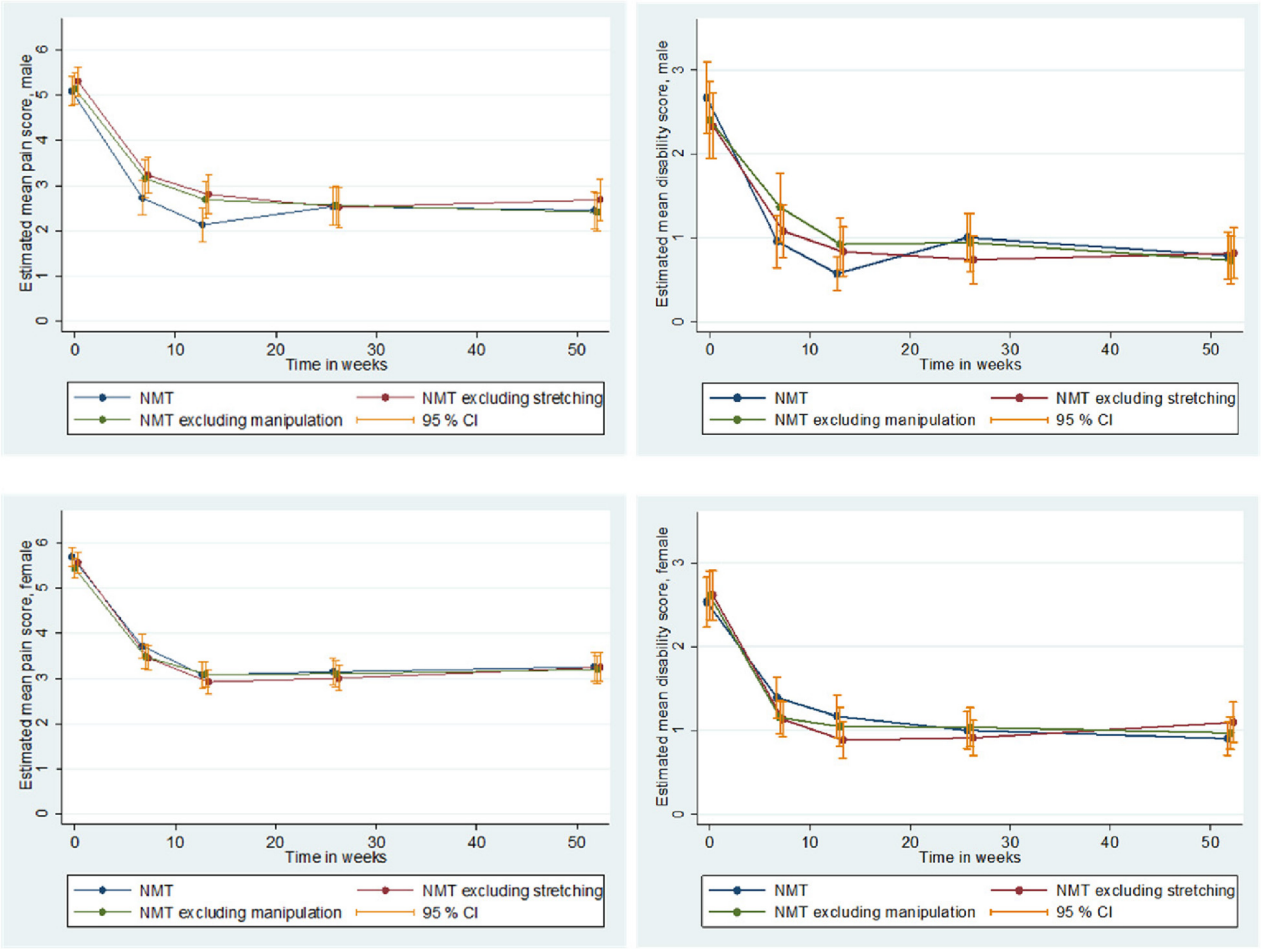
The mean scores of pain intensity and pain related disability. The mean scores of pain intensity and pain related disability with 95 % confidence intervals (95 % CI) for male and female patients at all follow-ups over one year

The joint test of difference – in the GEE framework taking repeated measurements at 7, 12, 26 and 52 weeks into consideration –showed no disparities between the treatment arms regarding mean score in pain intensity (*p* = 0.98; Wald test) and pain related disability (*p* = 0.88; Wald test) (not in table). Figure 3 shows the gender specific mean scores of pain intensity and pain related disability with 95 % CI at all follow-ups. No systematic differences in effect were seen when male and female patients were analyzed separately, especially not in the long term.

Concerning perceived recovery, in the GEE framework taking repeated measurements at 7, 12, 26 and 52 weeks into consideration –no differences in effect between the treatment arms were shown (*p* = 0.98; Wald test) (not in table).

The proportion that had not sought additional care for their neck/back pain the preceding three months at the 52-week follow-up was 61 % in all three treatment arms.

## Discussion

In this RCT with the aim of comparing the treatment effect of combinations of different treatment techniques within NMT for non-specific neck and/or back pain, no significant differences in effect between NMT (spinal manipulation, spinal mobilization, stretching and massage) and NMT excluding spinal manipulation and NMT excluding stretching respectively were found over 1 year concerning MCI in pain intensity and pain related disability or perceived recovery for patients seeking care. Neither were there significant differences in effect in gender stratified analyses. Furthermore, there were no clear differences between the treatment arms during the 1 year follow-up regarding care seeking for their back/neck pain; the proportion that had not sought additional care the presiding three months at the 12 months follow-up was the same (61 %) in all three treatment arms. The findings from this study are similar to findings in some previous studies [5, 30, 31]. In a recent Cochrane systematic review Gross et al. concluded that manipulation and mobilization provide similar effect for neck pain in mediate/short/intermediate-term follow-up [5]. One study included in the systematic review by Rubinstein et al. [12] was a smaller trial by Cleland et al. indicating that there is a better effect on pain and disability in low back pain patients from spinal manipulation than from spinal mobilization in short-term (4 weeks) and in disability in long term (6 months) [32].

### Strengths

The current study is a large randomized controlled trial (*n* = 1057) which is optimal for the research question addressed. We had a low attrition rate which was equally distributed between the treatment groups (Fig. 1). Furthermore we measured several potential prognostic factors that may have an impact on the prognosis of back and neck pain, and none of these factors were confounders in our study. The distribution of these factors was similar across treatment arms, indicating that the risk for unmeasured and residual confounding is low. A trained research assistant implemented the randomization, the data collection and the data input. She was also responsible for data quality management which was performed regularly during the study. All analyzes were done by a biostatistician who was not involved in the data collection or the randomization process. We therefore claim that the study has high internal validity.

The outcomes are clinically important and measured longitudinally with 1 year follow-up enabling to compare the long term effects for different treatment modalities. The total number of therapists in this study was 260. This increases the external validity by referring the effect of the treatment to the therapy itself rather than a specific set of therapists.

### Limitations

The therapists in this study were students at their seventh semester out of total of eight semesters, thus not experienced therapists. This may have an effect on the results. The students had treated patients, 2 days a week, under supervision during six semesters before the study started, but it is possible that the results could have been different if the patients had been treated by registered naprapaths with longer clinical experience and more skills in manual techniques. This may limit the possibility to compare the results from our study to previous studies and it may affect the external validity of the results. To explore this, we have compared the results from this study with one of our previous studies comparing NMT with the advice to stay active for patients with neck and/or back pain [18]. In the current study, the proportion of patients reaching MCI in pain intensity was 64 % and a MCI in disability was 42 % in the NMT-group at the 12 weeks follow-up. In the previous study the corresponding proportions were 69 and 73 % for the index group (NMT). There might be some differences between the two study populations, and the similarities in proportions of MCI in pain but not in pain related disability indicate that the result of this trial cannot be generalized to treatment given by experienced naprapaths.

This study was completed at an educational clinic. To confirm if the patients in this trial were comparable to patients visiting other naprapath clinics in Stockholm, we conducted a validation study. In this validation study the first included 111 patients in our trial study were compared to 97 patients seeking care for neck and/or back pain during the same time period, in three other clinics in the same geographical area. The result were that the patients in this trial were younger compared to patients visiting the other clinics (35.0 [SD 12.0] years and 42.0 [10.7] years respectively). The mean number of previous naprapathic treatments for the patients in the present study was 1.9 (SD = 0.9) compared to 2.5 (SD = 0.7) for those visiting other clinics. Additional parameters like physical activity, pain and disability scores and educational level were equal in the two populations (unpublished data by Joakim Ahlgren).

### Implication for clinical practice

There is an ongoing discussion about if treatment with some of the manual techniques used by manual therapists is associated with a higher risk of adverse events, and if such an increased risk is relevant for the potential benefits in effect of the treatment. The results of this study suggest that exclusion of spinal manipulation or stretching from combined manual therapy do not have a significant effect on improvement in pain intensity, in pain related disability and in perceived recovery in short and long term for patients seeking care for unspecific back and/or neck pain. In our previously published study [19] based on the first included 767 patients in the same trial, exclusion of spinal manipulation or stretching did not affect the risk of adverse events for patients with non-specific neck and/or back pain.

These findings might be of importance for patients and for manual therapists when choosing treatment strategies for non-specific neck and/or back pain, even though the result might be limited to treatments given by students. It might not be of importance if we use spinal manipulation and/or stretching strategies when we use combined manual therapy to treat unspecific back and/or neck pain on a group level, and therefore the choice of techniques can be based also on clinical and clinicians indications and patient preferences.

## Conclusion

The effect of manual therapy including spinal manipulation, mobilization, stretching and massage for male and female patients seeking care for neck and/or back pain at an educational clinic is similar regardless if spinal manipulation or stretching is left out as a treatment option.

## Ethics approval and consent to participate

The trial was approved by the Ethical review board in Stockholm, Sweden, 2009/1848-31/2. Informed consent was received from all study participants including consent for publication of the results.

## Consent for publication

Not applicable.

## Availability of data and materials

The dataset supporting the conclusions of this article is stored at the Institute of Environmental Medicine, Karolinska Institutet, Stockholm, Sweden, and the primary investigator is ES. Data will not be shared or available in an open access repository because the authors have not finished the data analysis yet. If anyone is interested in exploring specific issue, please contact ES.

## Abbreviations

CPQ: Chronic Pain Questionnaire
GEE: generalized estimating equations
MCI: minimal clinically important improvement
NMT: naprapathic manual therapy
RCT: randomized controlled trial
RR: relative risk
SCNMM: Scandinavian College of Naprapathic Manual Medicine
SNA: Swedish Naprapathic Association
Stockholm MINT trial: Stockholm Manual Intervention Trial
